# *N*-(2,3,4-Trifluoro­phen­yl)phthalimide

**DOI:** 10.1107/S1600536810024177

**Published:** 2010-06-30

**Authors:** Xian-Shu Fu, Xiao-Ping Yu, Wei-Min Wang, Fang Lin

**Affiliations:** a College of Life Sciences, China Jiliang University, Hangzhou 310018, People’s Republic of China

## Abstract

In the title compound, C_14_H_6_F_3_NO_2_, the benzene ring and the phthalimide ring system make a dihedral angle of 60.12 (7)°. Weak inter­molecular C—H⋯O and C—H⋯F hydrogen bonds are present in the crystal structure.

## Related literature

The title compound is a key inter­mediate in the synthesis of organic electro-luminescent materials, see: Han & Kay (2005 ▶). For the synthesis, see: Valkonen *et al.* (2007 ▶); Barchin *et al.* (2002 ▶). For related structures, see: Xu *et al.* (2006 ▶); Fu *et al.* (2010 ▶).
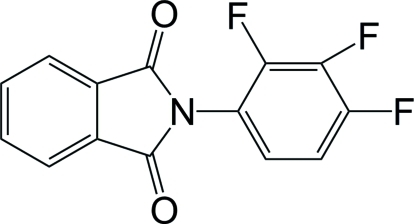


## Experimental

### 

#### Crystal data

C_14_H_6_F_3_NO_2_
*M* *_r_* = 277.20
Monoclinic, 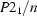

*a* = 6.8422 (14) Å
*b* = 21.082 (4) Å
*c* = 7.9727 (16) Å
β = 101.98 (3)°
*V* = 1125.0 (4) Å^3^
*Z* = 4
Mo *K*α radiation
μ = 0.14 mm^−1^
*T* = 113 K
0.20 × 0.18 × 0.12 mm

#### Data collection

Rigaku Saturn CCD area-detector diffractometer
Absorption correction: multi-scan (*CrystalClear*; Rigaku, 2005 ▶) *T*_min_ = 0.972, *T*_max_ = 0.983
8049 measured reflections
1980 independent reflections
1603 reflections with *I* > 2σ(*I*)
*R*_int_ = 0.034

#### Refinement

*R*[*F* ^2^ > 2σ(*F* ^2^)] = 0.034
*wR*(*F* ^2^) = 0.094
*S* = 1.03
1980 reflections
182 parameters
H-atom parameters constrained
Δρ_max_ = 0.34 e Å^−3^
Δρ_min_ = −0.21 e Å^−3^

### 

Data collection: *CrystalClear* (Rigaku, 2005 ▶); cell refinement: *CrystalClear*; data reduction: *CrystalClear*; program(s) used to solve structure: *SHELXS97* (Sheldrick, 2008 ▶); program(s) used to refine structure: *SHELXL97* (Sheldrick, 2008 ▶); molecular graphics: *SHELXTL* (Sheldrick, 2008 ▶); software used to prepare material for publication: *SHELXTL*.

## Supplementary Material

Crystal structure: contains datablocks I, global. DOI: 10.1107/S1600536810024177/ng2787sup1.cif

Structure factors: contains datablocks I. DOI: 10.1107/S1600536810024177/ng2787Isup2.hkl

Additional supplementary materials:  crystallographic information; 3D view; checkCIF report

## Figures and Tables

**Table 1:** Hydrogen-bond geometry (Å, °)

*D*—H⋯*A*	*D*—H	H⋯*A*	*D*⋯*A*	*D*—H⋯*A*
C4—H4⋯O2^i^	0.95	2.55	3.1855 (18)	124
C10—H10⋯F1^ii^	0.95	2.54	3.3647 (18)	145
C11—H11⋯O1^iii^	0.95	2.55	3.428 (2)	154

Symmetry codes: (i) 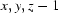; (ii) 
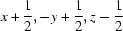
; (iii) 
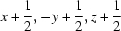
.

**Fig. 1.**
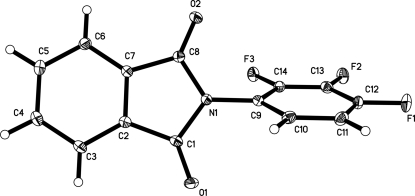
View of the molecule of showing the atom-labelling scheme. Displacement ellipsoids are drawn at the 30% probability level.

## supplementary crystallographic information

### Comment

The title compound is a key intermediate in the synthesis of organic electro-luminescent materials. The emission of light by organic molecules exposed to an electric field has been wide investigated in both an academic and industrial context. (Han & Kay, 2005).

The molecular structure of the title compound is illustrated in Fig. 1. In the title compound, two rings are nearly planar, but the molecule as a whole is not planar. The dihedral angle between the benzene ring and the phthalimide plane is 60.12 (7) °, which is similar to 59.95 (4) ° found in a related compound *N*-(2-fluorophenyl)phthalimide (Xu *et al.* 2006). Weak intermolecular C—H···O and C—H···F hydrogen bonding are present in the crystal structure (Table 1).

### Experimental

An acetic acid solution of phthalic anhydride (14.8 g, 100 mmol) and 2,3,4-trifluoroaniline (10.55 ml, 100 mmol) was refluxed overnight, and then filtered. The crude product was recrystallized from ethyl acetate.

### Refinement

H atoms were positioned geometrically and refined as riding with C—H = 0.95 Å, and *U*_iso_(H) = 1.2*U*_eq_(C).

### Crystal data

**Table:**

C_14_H_6_F_3_NO_2_	*F*(000) = 560
*M**_r_* = 277.20	*D*_x_ = 1.637 Mg m^−^^3^
Monoclinic, *P*2_1_/*n*	Mo *K*α radiation, λ = 0.71073 Å
Hall symbol: -P 2yn	Cell parameters from 3644 reflections
*a* = 6.8422 (14) Å	θ = 1.9–27.8°
*b* = 21.082 (4) Å	µ = 0.14 mm^−^^1^
*c* = 7.9727 (16) Å	*T* = 113 K
β = 101.98 (3)°	Prism, colorless
*V* = 1125.0 (4) Å^3^	0.20 × 0.18 × 0.12 mm
*Z* = 4	

### Data collection

**Table:**

Rigaku Saturn CCD area-detector diffractometer	1980 independent reflections
Radiation source: rotating anode	1603 reflections with *I* > 2σ(*I*)
confocal	*R*_int_ = 0.034
Detector resolution: 7.31 pixels mm^-1^	θ_max_ = 25.0°, θ_min_ = 1.9°
ω and φ scans	*h* = −8→8
Absorption correction: multi-scan (*CrystalClear*; Rigaku, 2005)	*k* = −24→25
*T*_min_ = 0.972, *T*_max_ = 0.983	*l* = −7→9
8049 measured reflections	

### Refinement

**Table:**

Refinement on *F*^2^	Secondary atom site location: difference Fourier map
Least-squares matrix: full	Hydrogen site location: inferred from neighbouring sites
*R*[*F*^2^ > 2σ(*F*^2^)] = 0.034	H-atom parameters constrained
*wR*(*F*^2^) = 0.094	*w* = 1/[σ^2^(*F*_o_^2^) + (0.0663*P*)^2^] where *P* = (*F*_o_^2^ + 2*F*_c_^2^)/3
*S* = 1.03	(Δ/σ)_max_ = 0.002
1980 reflections	Δρ_max_ = 0.34 e Å^−^^3^
182 parameters	Δρ_min_ = −0.21 e Å^−^^3^
0 restraints	Extinction correction: *SHELXL97* (Sheldrick, 2008), Fc^*^=kFc[1+0.001xFc^2^λ^3^/sin(2θ)]^-1/4^
Primary atom site location: structure-invariant direct methods	Extinction coefficient: 0.097 (6)

### Special details

**Table:**

Geometry. All e.s.d.'s (except the e.s.d. in the dihedral angle between two l.s. planes) are estimated using the full covariance matrix. The cell e.s.d.'s are taken into account individually in the estimation of e.s.d.'s in distances, angles and torsion angles; correlations between e.s.d.'s in cell parameters are only used when they are defined by crystal symmetry. An approximate (isotropic) treatment of cell e.s.d.'s is used for estimating e.s.d.'s involving l.s. planes.
Refinement. Refinement of *F*^2^ against ALL reflections. The weighted *R*-factor *wR* and goodness of fit *S* are based on *F*^2^, conventional *R*-factors *R* are based on *F*, with *F* set to zero for negative *F*^2^. The threshold expression of *F*^2^ > σ(*F*^2^) is used only for calculating *R*-factors(gt) *etc*. and is not relevant to the choice of reflections for refinement. *R*-factors based on *F*^2^ are statistically about twice as large as those based on *F*, and *R*- factors based on ALL data will be even larger.

### Fractional atomic coordinates and isotropic or equivalent isotropic displacement parameters (Å^2^)

**Table:**

	*x*	*y*	*z*	*U*_iso_*/*U*_eq_	
F1	0.36805 (13)	0.27803 (5)	1.30697 (12)	0.0324 (3)	
F2	0.04277 (12)	0.20200 (5)	1.25161 (11)	0.0286 (3)	
F3	0.00346 (11)	0.10870 (4)	1.01757 (10)	0.0245 (3)	
O1	0.29321 (15)	0.14544 (5)	0.58108 (12)	0.0246 (3)	
O2	0.28164 (15)	0.00092 (5)	1.01024 (12)	0.0228 (3)	
N1	0.28999 (17)	0.08556 (6)	0.82561 (14)	0.0181 (3)	
C1	0.28328 (19)	0.09456 (7)	0.64828 (17)	0.0176 (3)	
C2	0.26306 (18)	0.02990 (7)	0.57338 (17)	0.0162 (3)	
C3	0.2489 (2)	0.01067 (7)	0.40531 (18)	0.0193 (4)	
H3	0.2475	0.0406	0.3160	0.023*	
C4	0.23668 (19)	−0.05431 (8)	0.37222 (18)	0.0214 (4)	
H4	0.2272	−0.0690	0.2582	0.026*	
C5	0.2381 (2)	−0.09816 (8)	0.50331 (18)	0.0231 (4)	
H5	0.2302	−0.1422	0.4771	0.028*	
C6	0.2507 (2)	−0.07859 (7)	0.67177 (18)	0.0200 (3)	
H6	0.2510	−0.1083	0.7613	0.024*	
C7	0.26293 (18)	−0.01410 (7)	0.70366 (16)	0.0161 (3)	
C8	0.27847 (19)	0.02073 (7)	0.86743 (17)	0.0168 (3)	
C9	0.31396 (19)	0.13524 (7)	0.94859 (17)	0.0175 (3)	
C10	0.4794 (2)	0.17526 (7)	0.97522 (18)	0.0216 (4)	
H10	0.5796	0.1693	0.9102	0.026*	
C11	0.4998 (2)	0.22367 (8)	1.09549 (19)	0.0240 (4)	
H11	0.6125	0.2510	1.1129	0.029*	
C12	0.3538 (2)	0.23125 (7)	1.18896 (18)	0.0225 (4)	
C13	0.1879 (2)	0.19236 (8)	1.16429 (18)	0.0207 (4)	
C14	0.16934 (19)	0.14500 (7)	1.04425 (17)	0.0181 (3)	

### Atomic displacement parameters (Å^2^)

**Table:**

	*U*^11^	*U*^22^	*U*^33^	*U*^12^	*U*^13^	*U*^23^
F1	0.0371 (5)	0.0252 (6)	0.0336 (5)	−0.0042 (4)	0.0044 (4)	−0.0152 (4)
F2	0.0289 (5)	0.0319 (6)	0.0280 (5)	0.0003 (4)	0.0132 (4)	−0.0096 (4)
F3	0.0213 (5)	0.0273 (6)	0.0262 (5)	−0.0077 (4)	0.0080 (3)	−0.0059 (4)
O1	0.0342 (6)	0.0189 (7)	0.0220 (6)	0.0039 (5)	0.0086 (4)	0.0058 (4)
O2	0.0300 (6)	0.0236 (7)	0.0159 (5)	−0.0026 (4)	0.0072 (4)	0.0024 (4)
N1	0.0239 (6)	0.0158 (7)	0.0156 (6)	−0.0014 (5)	0.0062 (5)	−0.0003 (5)
C1	0.0159 (7)	0.0224 (9)	0.0150 (7)	0.0029 (6)	0.0044 (6)	0.0032 (6)
C2	0.0126 (6)	0.0191 (9)	0.0172 (7)	0.0018 (6)	0.0039 (5)	0.0014 (6)
C3	0.0164 (7)	0.0271 (10)	0.0145 (7)	0.0022 (6)	0.0030 (5)	0.0015 (6)
C4	0.0175 (7)	0.0288 (10)	0.0177 (7)	0.0014 (6)	0.0029 (6)	−0.0041 (6)
C5	0.0228 (8)	0.0215 (9)	0.0253 (8)	−0.0002 (6)	0.0053 (6)	−0.0065 (6)
C6	0.0210 (7)	0.0184 (9)	0.0209 (8)	0.0001 (6)	0.0047 (6)	0.0006 (6)
C7	0.0139 (7)	0.0190 (9)	0.0157 (7)	0.0007 (6)	0.0035 (5)	−0.0001 (5)
C8	0.0157 (7)	0.0179 (9)	0.0176 (7)	0.0003 (6)	0.0051 (5)	0.0010 (6)
C9	0.0216 (7)	0.0165 (9)	0.0141 (7)	0.0006 (6)	0.0027 (6)	0.0007 (6)
C10	0.0222 (7)	0.0198 (9)	0.0242 (8)	−0.0010 (6)	0.0076 (6)	0.0027 (6)
C11	0.0229 (8)	0.0199 (9)	0.0280 (8)	−0.0045 (6)	0.0025 (6)	0.0012 (6)
C12	0.0285 (8)	0.0165 (9)	0.0200 (7)	0.0011 (6)	−0.0003 (6)	−0.0041 (6)
C13	0.0215 (7)	0.0233 (9)	0.0181 (7)	0.0027 (6)	0.0058 (6)	0.0008 (6)
C14	0.0172 (7)	0.0180 (9)	0.0179 (7)	−0.0029 (6)	0.0011 (6)	0.0005 (6)

### Geometric parameters (Å, °)

**Table:**

F1—C12	1.3525 (17)	C4—H4	0.9500
F2—C13	1.3411 (17)	C5—C6	1.390 (2)
F3—C14	1.3486 (16)	C5—H5	0.9500
O1—C1	1.2072 (18)	C6—C7	1.382 (2)
O2—C8	1.2087 (16)	C6—H6	0.9500
N1—C8	1.4129 (19)	C7—C8	1.4824 (19)
N1—C1	1.4178 (17)	C9—C14	1.384 (2)
N1—C9	1.4207 (18)	C9—C10	1.392 (2)
C1—C2	1.483 (2)	C10—C11	1.387 (2)
C2—C3	1.3840 (19)	C10—H10	0.9500
C2—C7	1.3927 (19)	C11—C12	1.374 (2)
C3—C4	1.394 (2)	C11—H11	0.9500
C3—H3	0.9500	C12—C13	1.381 (2)
C4—C5	1.394 (2)	C13—C14	1.371 (2)
			
C8—N1—C1	111.90 (11)	C2—C7—C8	108.41 (13)
C8—N1—C9	123.66 (11)	O2—C8—N1	124.44 (13)
C1—N1—C9	124.39 (12)	O2—C8—C7	129.98 (14)
O1—C1—N1	124.63 (13)	N1—C8—C7	105.58 (11)
O1—C1—C2	130.33 (13)	C14—C9—C10	118.52 (13)
N1—C1—C2	105.04 (12)	C14—C9—N1	119.76 (12)
C3—C2—C7	121.07 (14)	C10—C9—N1	121.72 (12)
C3—C2—C1	129.87 (13)	C11—C10—C9	120.87 (13)
C7—C2—C1	109.05 (12)	C11—C10—H10	119.6
C2—C3—C4	117.42 (13)	C9—C10—H10	119.6
C2—C3—H3	121.3	C12—C11—C10	118.70 (14)
C4—C3—H3	121.3	C12—C11—H11	120.7
C5—C4—C3	121.26 (14)	C10—C11—H11	120.7
C5—C4—H4	119.4	F1—C12—C11	120.43 (13)
C3—C4—H4	119.4	F1—C12—C13	118.06 (13)
C6—C5—C4	121.12 (15)	C11—C12—C13	121.50 (14)
C6—C5—H5	119.5	F2—C13—C14	120.11 (13)
C4—C5—H5	119.5	F2—C13—C12	120.76 (13)
C7—C6—C5	117.30 (14)	C14—C13—C12	119.08 (13)
C7—C6—H6	121.3	F3—C14—C13	118.45 (12)
C5—C6—H6	121.3	F3—C14—C9	120.21 (12)
C6—C7—C2	121.82 (13)	C13—C14—C9	121.32 (13)
C6—C7—C8	129.77 (13)		
			
C8—N1—C1—O1	−179.01 (13)	C2—C7—C8—O2	179.32 (13)
C9—N1—C1—O1	−1.5 (2)	C6—C7—C8—N1	178.93 (13)
C8—N1—C1—C2	0.83 (14)	C2—C7—C8—N1	−0.75 (14)
C9—N1—C1—C2	178.32 (11)	C8—N1—C9—C14	−61.69 (18)
O1—C1—C2—C3	−0.5 (2)	C1—N1—C9—C14	121.12 (15)
N1—C1—C2—C3	179.72 (13)	C8—N1—C9—C10	118.90 (15)
O1—C1—C2—C7	178.52 (14)	C1—N1—C9—C10	−58.29 (18)
N1—C1—C2—C7	−1.30 (14)	C14—C9—C10—C11	0.3 (2)
C7—C2—C3—C4	−0.67 (19)	N1—C9—C10—C11	179.76 (13)
C1—C2—C3—C4	178.21 (12)	C9—C10—C11—C12	0.3 (2)
C2—C3—C4—C5	0.2 (2)	C10—C11—C12—F1	−179.77 (13)
C3—C4—C5—C6	0.3 (2)	C10—C11—C12—C13	−0.7 (2)
C4—C5—C6—C7	−0.34 (19)	F1—C12—C13—F2	1.9 (2)
C5—C6—C7—C2	−0.13 (19)	C11—C12—C13—F2	−177.27 (13)
C5—C6—C7—C8	−179.77 (13)	F1—C12—C13—C14	179.41 (13)
C3—C2—C7—C6	0.7 (2)	C11—C12—C13—C14	0.3 (2)
C1—C2—C7—C6	−178.43 (12)	F2—C13—C14—F3	−0.3 (2)
C3—C2—C7—C8	−179.64 (11)	C12—C13—C14—F3	−177.88 (12)
C1—C2—C7—C8	1.28 (14)	F2—C13—C14—C9	178.01 (12)
C1—N1—C8—O2	179.85 (12)	C12—C13—C14—C9	0.4 (2)
C9—N1—C8—O2	2.3 (2)	C10—C9—C14—F3	177.54 (12)
C1—N1—C8—C7	−0.09 (14)	N1—C9—C14—F3	−1.9 (2)
C9—N1—C8—C7	−177.60 (11)	C10—C9—C14—C13	−0.7 (2)
C6—C7—C8—O2	−1.0 (2)	N1—C9—C14—C13	179.83 (13)

### Hydrogen-bond geometry (Å, °)

**Table:**

*D*—H···*A*	*D*—H	H···*A*	*D*···*A*	*D*—H···*A*
C4—H4···O2^i^	0.95	2.55	3.1855 (18)	124
C10—H10···F1^ii^	0.95	2.54	3.3647 (18)	145
C11—H11···O1^iii^	0.95	2.55	3.428 (2)	154

Symmetry codes: (i) *x*, *y*, *z*−1; (ii) *x*+1/2, −*y*+1/2, *z*−1/2; (iii) *x*+1/2, −*y*+1/2, *z*+1/2.

## References

[bb1] Barchin, B. M., Cuadro, A. M. & Alvarez-Builla, J. (2002). *Synlett*, **2**, 343–345.

[bb7] Fu, X.-S., Yu, X.-P., Wang, W.-M. & Lin, F. (2010). *Acta Cryst.* E**66**, o1744. 10.1107/S1600536810023032 PMC3006924 21587961

[bb2] Han, K. J. & Kay, K. Y. (2005). *J. Korean Chem. Soc.* **49**, 233–238.

[bb3] Rigaku (2005). *CrystalClear* Rigaku Corporation, Tokyo, Japan.

[bb4] Sheldrick, G. M. (2008). *Acta Cryst.* A**64**, 112–122. 10.1107/S0108767307043930 18156677

[bb5] Valkonen, A., Lahtinen, T. & Rissanen, K. (2007). *Acta Cryst.* E**63**, o472–o473.

[bb6] Xu, D., Shi, Y.-Q., Chen, B., Cheng, Y.-H. & Gao, X. (2006). *Acta Cryst.* E**62**, o408–o409.

